# Evaluation of Web-Based Information on Spine Tuberculosis

**DOI:** 10.7759/cureus.28321

**Published:** 2022-08-23

**Authors:** Nilesh Barwar, Amit Sharma, Prem Prakash Sharma, Abhay Elhence

**Affiliations:** 1 Department of Orthopaedics, All India Institute of Medical Sciences, Bathinda, Bathinda, IND; 2 Department of Orthopaedics, All India Institute of Medical Sciences, Jodhpur, Jodhpur, IND; 3 Department of Community Medicine and Family Medicine, All India Institute of Medical Sciences, Jodhpur, Jodhpur, IND

**Keywords:** tb spine, bone tuberculosis, spine tuberculosis, pott’s paraplegia, pott’s spine

## Abstract

Objective: To evaluate and assess the information about spine tuberculosis available on the internet to the general public for its quality, adequacy, and authenticity.

Introduction: Tuberculosis is a bacteriological disease that has been associated with humankind since early human civilization. Spinal tuberculosis is an affection of the spine by the tubercle bacilli and can cause major complications, such as neurological dysfunction and spine deformities. Since the revolution in information technology, information about the disease is widely available on the internet.

Material and methods: A total of 68 websites were selected on Google, Yahoo, and MSN search engines for the information available about the disease. Scientific journals, books, and any other research materials were excluded from this study. The information was documented and evaluated for its validity, sufficiency, and authorship.

Results: The public education websites (PEdWebs: Those websites which did not have direct involvement in patient care) were the major source of the information with 58.82% (40) of it coming from them. In all, there were 69% (47) physicians and 30.9% (21) non-physicians. Among the physicians, 89.4% reviewed the information through commercial websites. “Inadequate” information about spine tuberculosis was provided by 11.8% (8), “Moderate information” by 51.5% (35), and “Sufficient information” by 36.8 % (25) of the websites. Among the websites, 13.2% (9) did not mention any specific presentation of the disease. Sufficient information on that aspect was provided by only 45.58% (31) of the websites. Only 39% emphasized the importance of early diagnosis and subsequent disease management. The majority, i.e., 79.4% (54), did not mention the preventive measures.

Conclusion: The internet has a lot of information regarding spine tuberculosis. The majority of this information comes from physicians. However, not every website has complete and essential information regarding the disease.

## Introduction

Tuberculosis (TB) is a pandemic and a major communicable human disease. The earliest human skeletal remains with the influence of TB date back to 8000-10000 years ago, corresponding to the Neolithic revolution, which came from the Near East [1]. In 2020, the Indian TB registry reported 18.05 lakh incident TB cases (new and relapse) with an ambitious target of notifying 29.9 lakh cases. Also, in 2019, 159 per 100,000 population were notified [2]. Worldwide, an estimate of 9.9 million people fell ill with TB in 2020, equivalent to 127 cases per 100,000 population. America has reported a rising number of cases of TB since 2016, primarily due to an increasing trend in Brazil. The 30 high TB burden countries account for 86% of all estimated incident TB cases worldwide, and eight of these countries account for two thirds of the global total: India (26%), China (8.5%), Indonesia (8.4%), the Philippines (6.0%), Pakistan (5.8%), Nigeria (4.6%), Bangladesh (3.6%), and South Africa (3.3%) [3].

TB can affect anyone, regardless of age or sex. The highest burden is in adult men, who accounted for 56% of all TB cases in 2020; by comparison, adult women accounted for 33% and children for 11% of the cases. Between 2018 and 2019, the annual rate of decline was 2.3% and 1.2% for the incidence rate and the absolute number of incident cases, respectively. Globally, the reduction in the number of TB deaths between 2015 and 2020 was only 9.2%. The two countries with the largest absolute reductions in TB notifications between 2019 and 2020, India and Indonesia, had previously been the main contributors to the large global increase in TB notifications between 2013 and 2019. Their combined annual total number of notifications had increased by 1.2 million in that period, but between 2019 and 2020, it fell by 0.7 million.

In 2020, of the 4.8 million people diagnosed with pulmonary TB in the world, 59% had bacteriologically confirmed cases. This was a slight increase from 57% (out of a total of 6 million) in 2019. Spine TB is an extension of the TB infection to the spinal column. It has potential complications of paralysis of extremities with deformity of the spine, including mortality. We searched the internet to analyze the information available about the spine TB for spreading awareness about this subject among people. Scientific journals, online books, and other literary materials were excluded from the study.

## Materials and methods

From December 2021 to April 2022, we evaluated all the information available about spine TB on the internet on three search engines (Google, Yahoo, and MSN). We searched the information regarding spine TB with various terms such as spine TB, Pott’s spine, and Pott’s paraplegia. Any scientific journal publications, online books, and literature on spine TB were excluded from the study. The information was sought with a viewpoint of how much and how accurate the information regarding spinal TB was available to the general population. It was then assessed with various parameters that were structured before starting the study. Only English language websites were considered in the study. Duplicated information was counted as one. The information was evaluated on the following 26 headings Table 1.

**Table 1:** Parameters Studied for the Evaluation of the Study TB: Tuberculosis

Serial No.	Parameters evaluated for the study
1	Authorship/source: private/public, news bulletin/stories, universities.
2	Charity organization
3	Updating the information
4	Information was given by a physician or non-physician
5	Commercial or non-commercial aspects of the websites
6	Any reference backing of the information, viz. peer reviewed or non-peer reviewed journals
7	Explanation of aetiology of spine TB
8	Mention of risk of mortality from the disease
9	Any description of preventive measures
10	Importance of early diagnosis
11	Description of primary organ (lungs or genito-urinary tract) affection)
12	Information on the prevalence of extra-pulmonary TB
13	Description of the prevalence of spine TB
14	Mode of transmission of the disease
15	Any risk factors for the disease
16	Description of constitutional symptoms
17a	Description regarding specific presentation of the disease: (1) no information, (2) neurological deficit, (3) spine deformity, (4) limb shortening, and (5) presentation with no specific signs and symptoms
18	Information regarding damage reversibility following spine TB
19	Type of treatment modality advised
20	Duration of medication
21	Specific indications of surgical intervention for the disease
22	Description of disease pathology by photos, videos and sketches etc.
23	Information about diagnostic test, laboratory tests & values etc.
24	Description regarding physical therapy.
25b	Adequacy of the information: (1) mode of transmission, (2) symptoms, (3) risk factors, (4) treatment modality, and (5) anatomical descriptions.
26	Contact information of the author/information provider

The information on the specific presentation included five elements as described in Table 1. The available information was graded zero or one under the following headings based on the presence of elements­­­: inadequate information, two pieces: moderate information, and three or more: sufficient information. The adequacy of information consisted of five elements as depicted in Table 1. The information was graded as per the number of responses from websites. Zero or one piece of information was classified as “Inadequate information,” two to three pieces of information as “Moderate,” and more than four as “Sufficient information.”

Those who uploaded the information were classified as physicians (if they held degrees/diplomas in the healthcare sector) and non-physicians (if they did not have degrees/diplomas in the healthcare sector). In total, 82 websites were found on the search engines mentioned. Ten of them were excluded from the study on account of duplication, and four were excluded due to the absence of meaningful information. Finally, 68 websites (33 from Google, 27 from Yahoo, and 8 from MSN) were included in the final evaluation.

The sources of information were categorized as (i) public education websites (PEdWebs) - the websites that did not have direct involvement in patient care, (ii) private (these included private hospitals, clinics, diagnostic laboratories, and private practitioners (iii) news bulletins or stories. All the information gained from the above headings/items was pooled and evaluated using SPSS software (SPSS Inc., Chicago).

## Results

Sources and reviewers of the information

PedWebs were the major source of the information at 59% (40) of the websites. Among them, 75% (32) had a commercial interest in providing the information and 65% (26) had physicians providing the information. In all, 69% (47) of the information sources were physicians and 31% (21) were non-physicians. About 90% of the physicians reviewed the information through commercial websites. Among the PedWebs, 43% (17) indicated surgery, and out of those, 58% (10) were authored by physicians.

Authorship of the information and source

Out of all the physicians, 58% authored the information through the PedWebs, 41% (19%) through private websites, and the rest, 4.2% (2), through news bulletins and stories.

Mentions of indications of surgery and the basis of information

Out of the 68 websites, 46% (31) indicated surgery as one of the modalities of treatment. The rest, 55% (37), did not show any such indication. Out of the websites that indicated surgery, 68 % (21) were authored by physicians and 26 % (10) by non-physicians. In 81% (55) of the cases, the information was based on experience, and in 19% (13) on peer reviews journals. Among the 55 persons who had authored information based on experiences, 67% (37) were physicians and 31% (18) were non-physicians.

Duration of medication for the disease

Out of all the websites, 60.3% (41) contained no information regarding the duration of medication, while 20.6% (14) mentioned medication duration as ≤1 year and 19% (13) as >1 year. Out of the 41 that did not mention regarding medication, 51% (24) were authored by physicians.

Adequacy of the information regarding Spine TB

**Figure 1:**
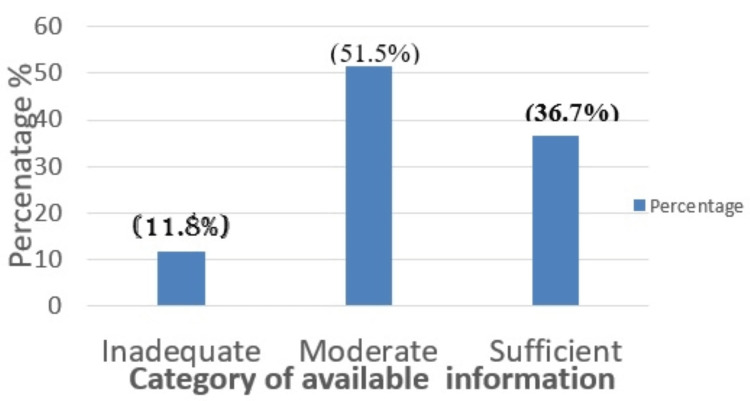
Adequacy of the Information Available About Spine TB

The adequacy of information was judged by five parameters, as described earlier. Approximately two-thirds of the websites provided inadequate to moderate information, and only about a third of them provided sufficient information (Figure 1).

The anatomical description was mentioned by 83.82% (57), both symptoms and treatment modality by 76.47% (52), and risk factors by 58.82% (40) of the websites. The mode of transmission was least frequently mentioned, only by 25% (17) of the websites.

Specific presentation of the disease

**Figure 2:**
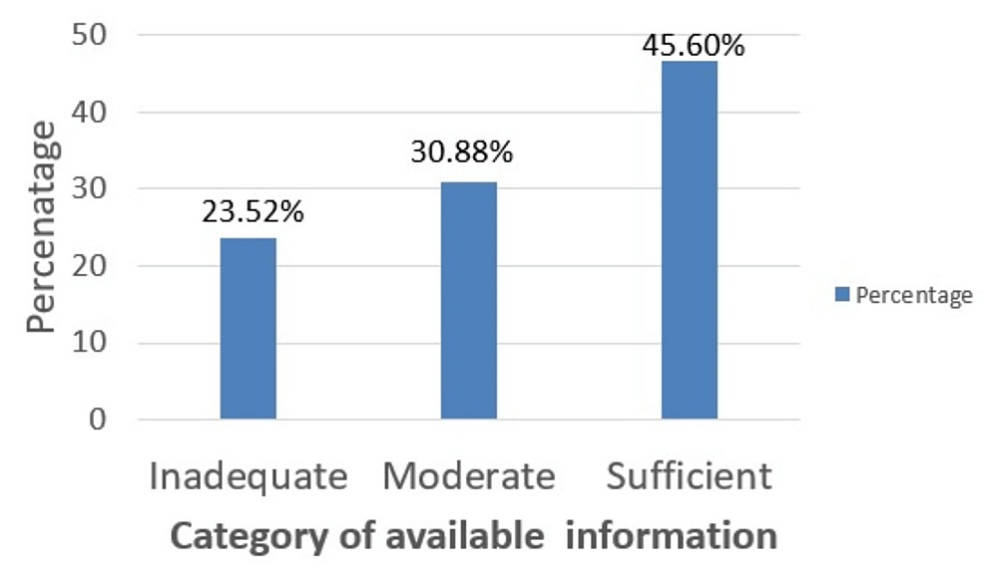
Available Information about Specific Presentations of Spine TB

The information regarding the specific presentation of the disease was evaluated under the following headings using five parameters, as described earlier. The majority of the websites, 85% (58), mentioned neurological deficit as a specific presentation, followed by 76% (52) mentioning deformities. Limb shortening was mentioned by 43% (29) of the websites, and 15% (15) of the websites mentioned no specific presentation. A small percentage, 13% (9), did not mention any specific presentation. Only about half of the websites, 46% (31), provided “sufficient” information on specific presentation of spine TB (Figure 2).

Mention of the aetiology of the disease

A majority of the websites, 58.8% (40), displayed the aetiology of the spine TB. Among the 47 websites authored by physicians, 57.4% (27) mentioned aetiology.

Treatment of the disease and preventive measures

**Figure 3:**
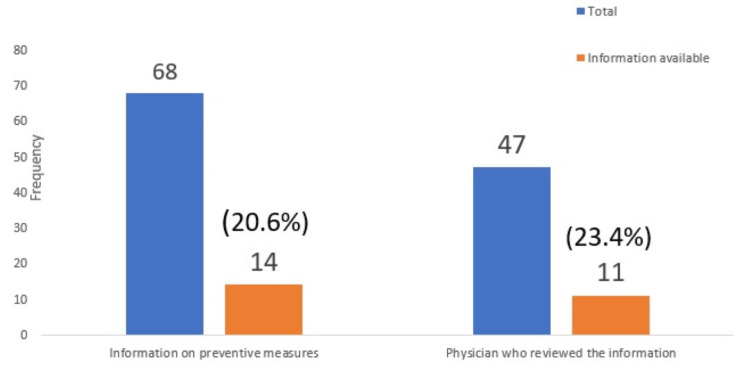
Information Available on Preventive Measures of TB

Most websites, 70.5 % (48), mentioned the treatment modality as both medical and surgical. A majority of the physicians, 72.3% (34), also advised the treatment modality as both. In contrast, with regard to the preventive aspect of the disease, 79.4% (54) of the websites did not mention any measures, and 77% of those authored by physicians also did not mention it (Figure 3).

Reference to peer-reviewed journals

Among the total, only 19% (13) of the websites gave detail regarding the reference to peer-reviewed journals. Out of these, 76.93% (10) were authored by physicians. 

Information regarding the prevalence of TB

Out of all the websites, 63.2% (43) did not mention the prevalence of spine TB. Even among the 47 websites authored by physicians, 59.6% (28) did not mention about the prevalence.

## Discussion

Availability and easy access to the internet has increased the availability of health information to the general public, which was difficult in the past. Today, more and more people seek information regarding health issues online and make informed decisions for their future treatment courses. In 2019, 53% of EU citizens, i.e., one in every two, aged 16-74 reported that they/ seek online health information, such as information related to injuries, diseases, nutrition, and improving health [4].

TB is caused by a bacterium called “*Mycobacterium tuberculosis*,” which usually spreads through the respiratory system. It chiefly attacks the lungs, and subsequently, any part of the body. The time of acquisition of TB infection and subsequent development of TB disease is variable, ranging from months to decades. Obtaining information about TB’s aetiology and management is now easier. As of January 2021, the Internet had 4.66 billion active users worldwide, i.e., 59.5% of the world population. Of these, 92.6% access the Internet via mobile devices [5].

In an online study on minimally invasive spine surgery (MISS), out of 150 websites, 50% of the authors were private physicians or from clinics. Most resources, 84%, (126), mentioned some benefits of MISS, but only 17% (26) described the risks of the procedure [6]. In another web-based study on minimally invasive total knee replacement, out of 150 websites, 51% were authored by hospitals/universities and 26% by private medical groups. Among the websites, 25% described the risks, and more than 82 % made specific claims regarding the advantages of the minimally invasive procedure. However, references to peer-reviewed journals were low at 3% [7].

In our study, the information provided by the 68 websites might be trusted in the majority (≈70%), where the information has been provided by physicians who had experience in health care. In contrast, the remaining one-third did not have health care professionals as their authors and hence it is advisable to exercise some caution while interpreting them. Our study found that instead of private websites, PEdWebs were at the forefront in providing information regarding spine TB, though their information was inadequate the majority of times (75%) with a commercial interest in form of product advertisement.

As TB is a bacteriological disease, chemotherapy is the mainstay of the management; many websites (≈90%) mentioned medical management with or without surgical procedures. In spine TB, some patients such as those with neurological deficits, spinal deformities, and instabilities may require surgical interventions [8]. Internet surfing lets people know that some spine TB cases (50% in the present study) need surgical invention for the disease’s management for some specific indications. In skeletal TB, the WHO recommends nine months of chemotherapy with two months of intensive phase and seven months of continuation phase [9], whereas some authors had mentioned the duration of the medication from six months to two years [10]. In the present study, a majority of them (about 60%) did not describe the duration of treatment, even though this information is crucial for the treatment of the disease.

Regarding the mode of transmission, symptoms, risk factors, treatment modalities, and anatomical description for spine TB, the majority (51.5%) of the websites had only a moderate amount of information, and only a third displayed sufficient information. Information about these factors is insufficient on the internet. Spine TB is known to present with some specific presentations, such as neurological deficits, deformities, limb shortening, and sometimes with no specific presentations, too. Knowledge of specific presentations is crucial for patients, as this is a clue about the disease. Only around half of the websites provided “sufficient” information. Neurological deficit and deformity of the spine were the most frequently (85% each) described specific symptoms.

TB is commonly known as an infectious disease, and hence, the majority of the websites (about 60%) described the disease aetiology. TB is a disease of poverty and opportunistic infection. Low-living standards and poor hygiene promote the risk of infection [11]. Yet, 80% of websites in the study did not mention this. Moreover, 64% of the websites did not even mention the prevalence of the disease in society. Skeletal TB occurs by a hematogenous transmission of the bacilli from some primary sites, such as lungs, genito-urinary system, and lymph nodes. Also, almost 40% of the Indian population is estimated to have latent TB infection [11]. The development of the disease depends on the immune status, existing comorbidity, and risk factors [12]. In the present study, only half of the websites mentioned primary organ affection and the risk factors for disease development.

**Figure 4:**
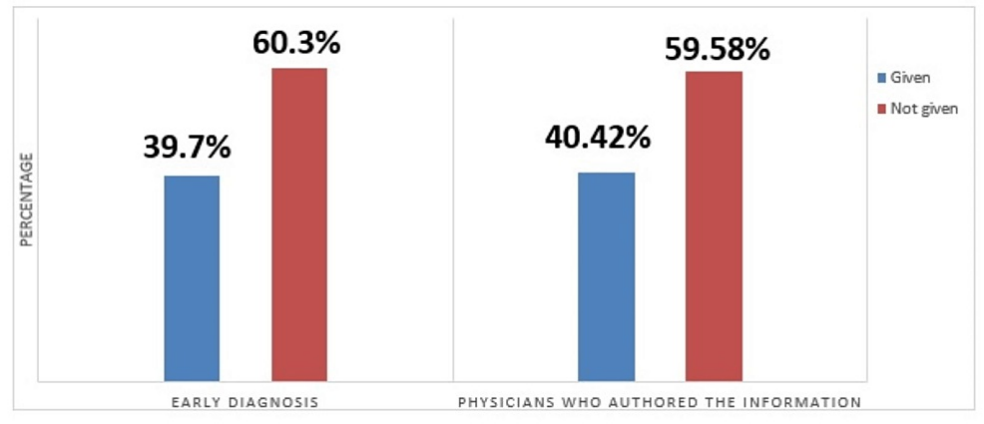
Information about the Importance of Early Diagnosis of Spine TB

The introduction of medication in the pre-destructive phase of the disease can reverse the damage and may lead to complete healing of the lesion without much deformity and neurological complication [13]. However, in the present study, the websites did not give much emphasis to the importance of early diagnosis and subsequent management (Figure 4).

## Conclusions

Tuberculosis is a major human menace. A plethora of information about spine TB is available on the internet. Most of this information on different websites come from the experiences of the authors and little of it is scientifically based. However, two-thirds of these authors are related to the health care sector in some way. Not every website has complete information about the disease. The quantum of information on the specific presentation of the disease as well as details on spine TB is not sufficient. In addition, information about the preventive measures and early diagnosis is minimal. Information regarding indication of surgical measure for the disease and the duration of drug therapy is also scarce. It is suggested that a patient should not rely on a single website for the treatment, and it is strongly advised for people to visit a physician for holistic information regarding the ailment for optimum management of the disease.
